# Application of operant demand in autism and developmental disabilities: An updated review (2017–2025)

**DOI:** 10.1002/jaba.70090

**Published:** 2026-10-13

**Authors:** Ji Young Kim, Lesley Shawler, Shawn Gilroy

**Affiliations:** ^1^ School of Behavioral Sciences and Education Pennsylvania State University–Harrisburg Middletown PA USA; ^2^ School of Psychological and Behavioral Sciences Southern Illinois University Carbondale, Carbondale IL USA; ^3^ Department of Psychology Louisiana State University Baton Rouge LA USA

**Keywords:** autism, behavioral economics, developmental disabilities, operant demand, progressive ratio

## Abstract

Operant demand within behavioral economics provides a quantitative framework for evaluating reinforcer efficacy in applied research involving individuals with autism and those with related developmental disabilities. This systematic review extended the work of Gilroy, Kaplan, and Leader (2018) by examining applied operant demand research published between March 2017 and December 2025. Following PRISMA guidelines, 30 articles (31 experiments; 307 participants) were identified and coded for procedural features and methods, including operant arrangements, demand and work output analyses, open and closed economies, and unit price applications. Results indicated increased use of demand and work output analyses and unit price applications. However, open and closed economies remained rarely evaluated and substantial variability was observed in procedural methods used to manipulate price. Findings indicate growing use of operant demand analyses for treatment durability and schedule thinning but continued underuse for generalization variables.

Behavioral interventions for individuals with autism spectrum disorder and those with developmental disabilities depend on identifying effective and durable reinforcers (Fisher et al., 1992; Pace et al., 1985), as access to meaningful reinforcers is critical for skill acquisition, reducing challenging behavior, and maintaining treatment gains. Although various preference assessments and reinforcer evaluations have been developed, these procedures may not always predict how reinforcers perform when effortful or sustained responding is required, which is often the case in real‐world interventions (DeLeon et al., 1996; Tustin, 1994).

Operant demand within behavioral economics offers a promising framework for addressing these challenges by extending analyses of reinforcers into domains traditionally associated with microeconomics. Central concepts within the operant demand framework (e.g., demand and work output functions, elasticity, unit price, open and closed economies, and substitutability) provide quantitative, concrete methods for evaluating how reinforcer value changes in response to effort, cost, or the presence of alternatives (Hursh, 1980, 1984; Hursh & Silberberg, 2008). A demand function shows how consumption (i.e., the quantity of a reinforcer obtained or consumed at a given price) decreases as its “price” (i.e., response effort or schedule requirement) increases, whereas a work output function reflects the amount of responding an individual will expend to maintain baseline levels of reinforcer consumption as its “price” increases. For example, a child with autism participating in a classroom program may initially complete one math problem to earn a break; as the requirement increases, total responding first increases (the child completes more problems overall to defend break consumption) and then decreases once demand becomes elastic. The peak of this work output function is O_*MAX*_, the greatest total responding emitted, and the price at which it occurs is P_*MAX*_. Elasticity refers to the sensitivity of consumption to changes in price, with inelastic demand indicating persistent or escalating work in response to increasing prices and elastic demand indicating reductions in work as a result of increasing prices.

These relations also depend on the broader context, or economy type, in which reinforcers are available: In a closed economy, access to a reinforcer is restricted to the arranged contingencies and cannot be obtained elsewhere, whereas in an open economy, the same reinforcer (or alternatives) can be obtained outside those contingencies. For example, access to breaks at school is typically part of the regular classroom routine and available from multiple sources, reflecting an open economy. In contrast, access to a specific reinforcer (e.g., play with a preferred clinician) available only contingent on task completion within treatment sessions reflects a closed economy. Within such economies, reinforcers may interact through reinforcer–reinforcer relations such that consumption of a reinforcer increases as the price of another increases (i.e., substitutability). For example, if the price of spending time with a preferred clinician increases (e.g., requiring completion of 15 math problems), a child with autism might instead choose to spend time with another clinician who requires fewer math problems (e.g., requiring completion of five math problems). Collectively, these concepts characterize how reinforcer efficacy shifts across contexts, enabling researchers and practitioners to move beyond static measures of preference toward dynamic assessments that better reflect treatment‐relevant conditions. Table 1 includes a summary of these terms and definitions, and Figure 1 shows a visual display of demand and work output curves.

**TABLE 1:** Operant demand terminologies and definitions.

Term	Definition
Consumption	The quantity of a reinforcer obtained or consumed at a given price. Depending on the study, consumption may be measured as the number of reinforcers earned, items consumed, duration of access to a reinforcer, or another unit reflecting reinforcer acquisition.
Demand function	A representation of how consumption of a reinforcer decreases as its price (i.e., response effort or schedule requirement) increases.
Work output function	A representation of the amount of responding an individual is willing to allocate to maintain baseline levels of reinforcer consumption as costs increase.
Elasticity	The sensitivity of reinforcer consumption to changes in price; inelastic demand indicates persistence despite rising costs, whereas elastic demand reflects greater reductions in consumption as costs increase.
Unit price	The cost of a reinforcer expressed relative to its magnitude (e.g., responses per second of access), permitting standardized comparisons across reinforcers and schedules.
Open and closed economies	Contexts in which reinforcers are available. In a closed economy, access is restricted to the arranged contingencies; in an open economy, the same reinforcer or alternatives may be available outside those contingencies.
Substitutability	A reinforcer–reinforcer relation in which consumption of one reinforcer increases as the cost of another rises, reflecting the functional replacement of reinforcers within an economy.
O_*MAX*_	The maximum response output emitted to obtain a reinforcer, typically measured as the highest number of responses allocated to a commodity across prices. O_*MAX*_ represents the point at which responding is greatest before further increases in price result in reduced responding.
P_*MAX*_	The price at O_M*AX*_. P_*MAX*_ represents the transition from inelastic to elastic demand and identifies the price at which response output is maximized.
Breakpoint	The price at which consumption reaches zero (i.e., the first price at which the individual stops responding for the reinforcer).
Demand intensity	The level of consumption when the price of a reinforcer is absent or minimal (i.e., free or near‐free access). Demand intensity reflects the amount of a reinforcer consumed before increases in price begin to influence behavior.

**FIGURE 1:**
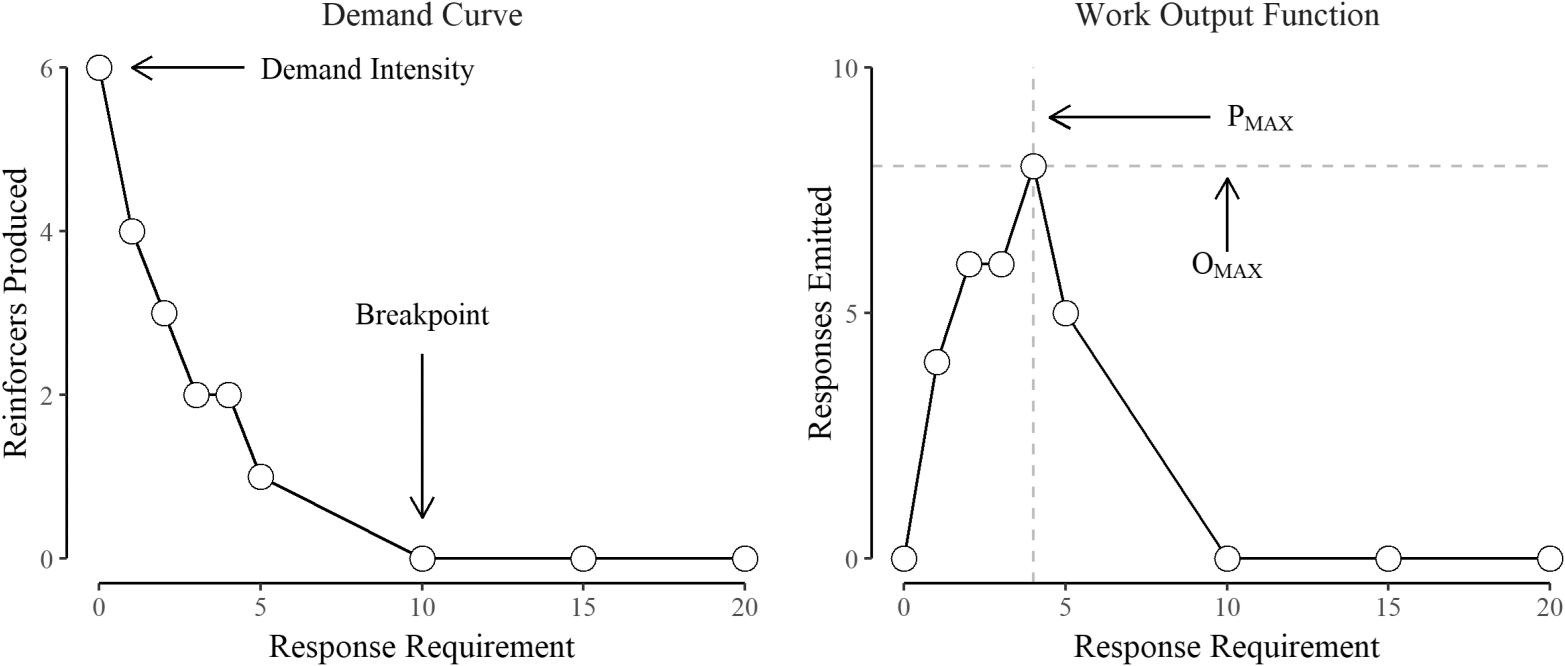
Example demand and work output curves.

Oftentimes, treatment outcomes for individuals with autism and those with developmental disabilities may depend on the persistence of reinforcer effects under conditions of increasing cost or constraint (Reed et al., 2013). In other words, treatment success may depend on how effective a reinforcer is as work requirements or response effort increase. To illustrate, reinforcement during schedule thinning is systematically delivered following progressively greater response requirements or longer delays (e.g., Hagopian et al., 2011; Kranak & Brown, 2024). Here, treatment success depends not merely on whether a reinforcer is initially effective but on whether its effects endure as work requirements or response effort increase. Despite the promise of operant demand to advance the assessment and treatment of behavioral concerns among individuals with autism and those with developmental disabilities, relatively little work has directly evaluated its application.

To better understand the extent of translation, Gilroy, Kaplan, and Leader (2018) conducted a systematic review to determine which operant demand concepts had been applied in assessment and treatment research involving individuals with autism and those with developmental disabilities through early 2017. Their review identified 22 studies, most of which relied on progressive‐ratio (PR) schedules (i.e., systematically increasing the number of required responses to earn a reinforcer after each delivery; Roane, 2008) to evaluate reinforcer efficacy. Concepts with strong potential to inform assessment and treatment decisions, including demand curves, open versus closed economies, and unit pricing, were observed only rarely. Since that review, operant demand research in these populations has continued to expand, supported in part by advancements in technology and methodological preparations that make economic analyses more feasible in applied settings (Gilroy, Kaplan, Reed, et al., 2018; Kaplan et al., 2019). For example, studies have examined token economies to optimize reinforcement schedules for children with autism (Levins & Gilroy, 2025), assessed the persistence of reinforcer value in children with autism (Gilroy et al., 2021), and investigated how reinforcer value affects persistence and bursting during extinction in adults with developmental disabilities (Lambert et al., 2024). Furthermore, analyses of demand intensity and elasticity (i.e., measures of the reinforcing efficacy of a stimulus) may clarify why certain reinforcers maintain responding in structured programs but not in naturalistic environments or why treatment durability varies as schedules of reinforcement are thinned (Bonner et al., 2022).

The purpose of this review is to extend Gilroy, Kaplan, and Leader (2018) by updating the literature through December 2025 and examining how applied research on operant demand has been incorporated into assessment and treatment research involving individuals with autism and those with related developmental disabilities. Throughout this review, references to the assessment and treatment of autism and developmental disabilities refer to the assessment and treatment of behavioral challenges, skill acquisition, and reinforcement processes commonly addressed within these populations rather than to the treatment of diagnostic labels themselves. This review addresses three questions: (1) Which operant demand concepts and procedures have been evaluated in the assessment and treatment literature involving individuals with autism and those with developmental disabilities? (2) In which assessment and treatment areas and contexts have these concepts been applied? (3) Which populations are most commonly included in applied operant demand research? The review also highlights key gaps in the application of operant demand concepts by identifying areas where important concepts remain underexplored.

## METHOD

The literature search adhered to the Preferred Reporting Items for Systematic Reviews and Meta‐Analyses (PRISMA) statement (Page et al., 2021). The methods to identify, screen, include, and code articles were specified in advance and registered through PROSPERO#: CRD42023479981 in November 2023. Figure 2 depicts the identification, screening, and eligibility process for the number of studies included.

**FIGURE 2:**
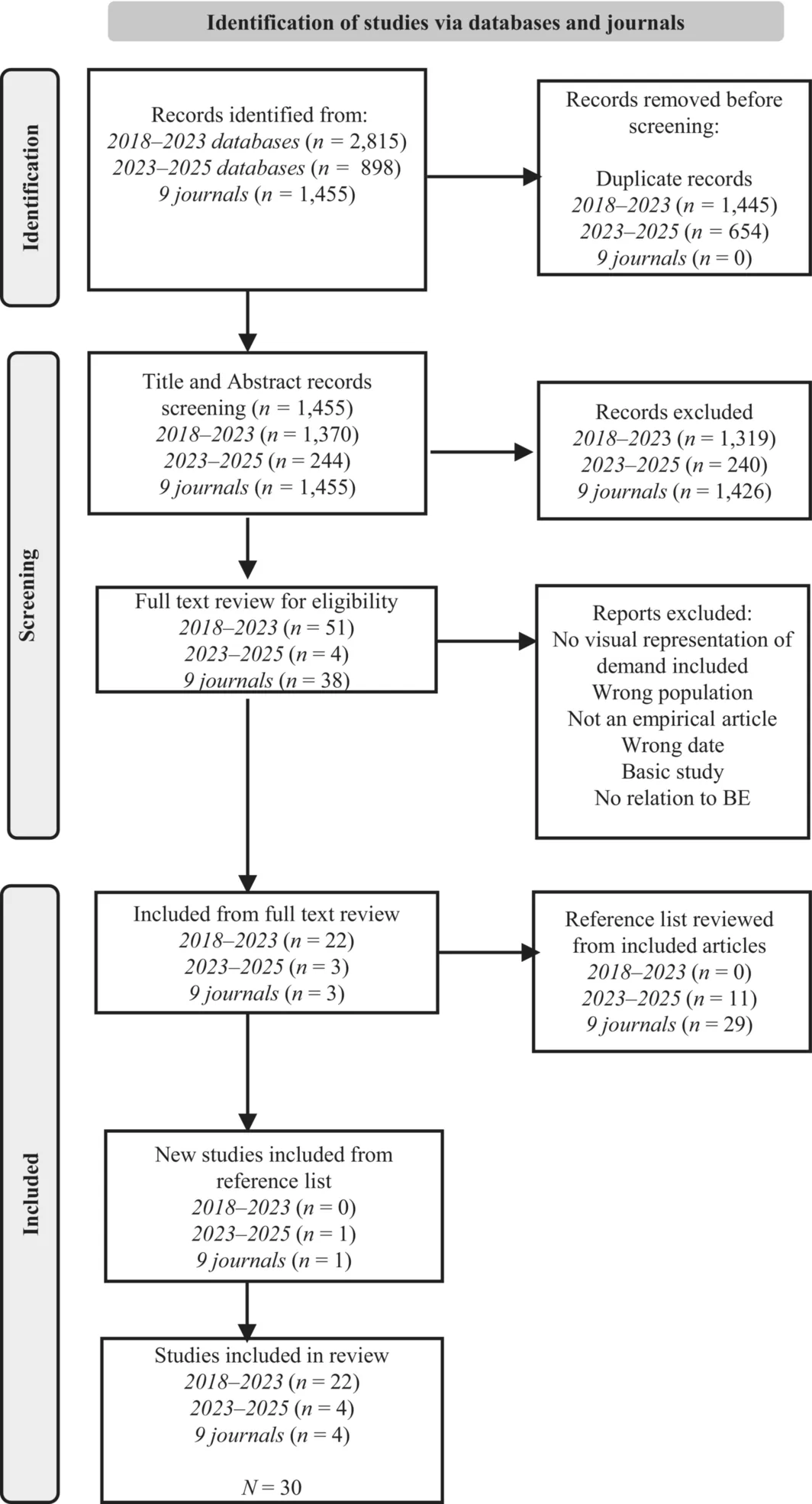
PRISMA flowchart of article search and identification process. BE = behavioral economics.

### Search and article identification

The first and second authors conducted an initial systematic search of the literature on studies targeting operant demand concepts with individuals with autism or those with developmental disabilities in December 2023 and January 2024. Specifically, we conducted a search through the same databases used by Gilroy, Kaplan, and Leader (2018) including SpringerLink, ScienceDirect, EBSCOhost, PubMed, and Scopus between March 2017 and October 2023. We chose these dates to extend the previous search parameters from Gilroy, Kaplan, and Leader, who searched through February 2017. We searched the same keywords used in Gilroy, Kaplan, and Leader, including Boolean operators and truncation: “progressive ratio,” “demand curve,” “open economy,” “closed economy,” and “unit price.” For each of these terms, we searched for it with AND “autis*” OR “disabilit*.” We then uploaded the relevant search lists into the free online systematic review platform Rayyan (Ouzzani et al., 2016) to complete the title and abstract screening.

To update the review beyond the initial search period, the same search procedures were replicated in April 2026 to identify studies published between November 2023 and December 2025. In addition, we implemented a targeted hand‐search procedure across nine journals that had published articles identified in the initial search of the current review: *American Journal on Intellectual and Developmental Disabilities, Autism, Behavioral Interventions, Behavior Modification, Developmental Neurorehabilitation, Education and Treatment of Children, Journal of Applied Behavior Analysis, Journal of Abnormal Child Psychology*, and *Journal of Behavioral Education*. All issues and volumes published between November 2023 and December 2025 in each journal were inspected. This hand‐search procedure was conducted to identify recently published articles that had not yet been indexed within the electronic databases at the time of the search.

### Inclusion and exclusion criteria

Inclusion criteria consisted of peer‐reviewed research articles published in English between March 2017 and December 2025 that had their full text available. Studies had to evaluate concepts and topics related to operant demand, including reinforcer efficacy procedures (PR schedules, reinforcer efficacy), demand functions, work output functions (with or without elasticity metrics), open and closed economies, or unit pricing logic (as either a primary or supplemental component). Studies using PR schedules had to include a visual representation of the demand or work output function curves (see Figure 1 for an example) to distinguish them from studies exploring relative reinforcing efficacy, which involve more dichotomizing price scaling to high and low levels rather than demand (i.e., how consumption or work varied across multiple schedules). Studies could use either a single‐case or group design, could be applied or translational in nature, and needed at least one participant who had diagnoses related to autism or developmental disabilities.

We excluded studies based on behavioral economic topics that were not included in the original review (e.g., matching law, delay discounting), studies using PR schedules without a visual representation, single‐case designs that did not use a rigorous experimental design with replication (i.e., AB design, pre/post), studies with nonhuman subjects (e.g., rats, pigeons), participants who had pathologies inconsistent with developmental disabilities (e.g., substance use, gambling), nonempirical articles (e.g., literature reviews, conceptual papers), and articles that were not published in English or in a peer‐reviewed journal.

### Screening and eligibility

Once articles were identified through title and abstract screening, we conducted a full‐text review to assess eligibility. After the title and abstract review, the authors used Rayyan's (https://www.rayyan.ai/) feature to remove all duplicates before starting the full‐text review. We then completed a full‐text review of each article to determine which articles met the inclusion criteria. After the full‐text review, the first and second authors reviewed the reference lists of each selected article to identify whether any additional studies met the inclusion criteria.

### Article coding

The first author reviewed all articles and coded 16 variables, including eight primary and eight secondary variables, using a customized data collection instrument created in Microsoft Forms. All variables were coded at the participant level, allowing each participant's data to be recorded. If a study included participants with and without disabilities, we included only those with a reported developmental disability diagnosis (e.g., Hangen et al., 2023). For studies using a group design, we reported data at the study level, as the authors reported them (e.g., Lloyd et al., 2021), as in Gilroy, Kaplan, and Leader (2018). Specifically, we selected each reported diagnosis for all participants and recorded the age range for all participants in the group. We reported data only for the group(s) that met the inclusion criteria (i.e., data for other groups were not reported). One study applied operant demand concepts to only one of its four participants; therefore, only data from that participant (Noah: Kastner et al., 2025) were included in our analysis. The specific primary variables included the presence of single‐ or concurrent‐operant arrangements, demand functions, work output functions, closed or open economies, and unit pricing and the procedural method including how the price was manipulated, whether the price manipulation occurred within or across sessions, and progression type (i.e., arithmetic, geometric, or arbitrary). The other eight variables included information related to the participant, including (a) diagnosis, (b) age, and (c) race, and experiment information including (d) dependent variables (e.g., task completion, challenging behavior, academic responses, simple‐operant task responses, preference selection, or other), (e) primary purpose of the study (i.e., to either increase functional skills or decrease challenging behavior), (f) whether the study was an assessment or treatment, (g) the intervention that was implemented (if applicable), and (h) the overall outcome (i.e., efficacious or effective). Table 2 provides the operational definition of each variable.

**TABLE 2:** Operational definition of variables coded in the review.

Variable	Definition
Single‐ or concurrent‐operant arrangement	In PR assessments, reinforcers can be evaluated either individually (i.e., single‐operant arrangements in which only one reinforcer is available and assessed in isolation) or along with other reinforcers (i.e., concurrent‐operant arrangements in which two or more reinforcers are available at the same time). We noted the number of reinforcer assessments conducted in single‐operant/concurrent‐operant arrangements within a study. For example, if a study included two single‐operant arrangements and no concurrent‐operant arrangements, we noted “2/0.“
Demand function	If a study visually displayed demand curve(s) (with or without elasticity metrics), we noted “Yes.” If none, we noted “‐.”
Work output function	If a study visually displayed work output function(s) (with or without elasticity metrics), we noted “Yes.” If none, we noted “‐.”
Closed or open economy	If a study investigated the concept of open and closed economies, we noted “Yes.” If none, we noted “‐.”
Unit pricing	If a study included unit‐pricing logic as either a primary or supplemental component, we noted “Yes.” If none, we noted “‐.”
Price manipulation method	We identified the specific procedural method for price manipulation used in each experiment and categorized them into one or more of the categories below using the definition listed: **Increasing FR requirement**: Systematic increases in the FR response requirement across price points. **Increasing VR requirement**: Systematic increases in the VR response requirement across price points. **Increasing token‐production schedule requirement**: Systematic increases in the response requirement required to earn a token within a token economy. **Increasing exchange‐production schedule requirement**: Systematic increases in the number of tokens required before access to the backup reinforcer became available. **Increasing token‐exchange schedule requirement**: Systematic increases in the token cost of obtaining a backup reinforcer within a token economy.
Within versus across sessions	If a study manipulated price within a session, we noted “Within session.” If a study manipulated price across sessions, we noted “Across sessions.”
Progression type [Step size]	We noted the specific progression type and step size of each price manipulation procedure. We categorized each price manipulation procedure into arithmetic (i.e., increase by adding a value in each trial/session; e.g., 1, 2, 3, 4 …), geometric (i.e., increase by multiplying a value in each trial/session; e.g., 1, 2, 4, 8 …), or arbitrary (neither arithmetic nor geometric) progression. We also noted the step size or the numerical increment by which the number of required responses increases after each reinforcer is earned.
Participant diagnoses	We noted each participant's specific type(s) of diagnosis or diagnoses.
Participant demographics	We noted the race of the participants (e.g., White, Black, Hispanic, Asian, multiracial). If this information was unavailable, we noted “missing information.”
Age	We noted each participant's age or a range if not reported.
Dependent variables	We identified the primary dependent variable(s) for each experiment and categorized them into one or more of the categories below using the definition listed: **Task completion:** Engagement in a response for a predetermined period or completing multiple steps of instructions or tasks (e.g., sequence of tasks, steps to be completed, or within a certain time frame). **Challenging behavior:** A pattern of behavior interfering with an individual's learning or engagement with others. **Academic responses:** Count of discrete responses in an academic context (e.g., math worksheet). **Simple‐operant task responses:** Single‐step, discrete responses in a nonacademic context. **Preference selection:** Selection of a preferred option out of an array of alternatives in a concurrent‐operant arrangement. **Other:** Dependent variable(s) that were not included in the aforementioned codes.
Decreasing challenging behavior versus increasing functional skills	We identified the primary purpose of the experiment and noted each as either decreasing maladaptive behavior, increasing functional skills, or neither. The purpose of an experiment was to decrease challenging behavior if the experiment focused on decreasing or eliminating the frequency, rate, duration, or intensity of the target behavior. The purpose of an experiment was to increase functional skills if an experiment focused on increasing frequency, rate, duration, or intensity of the target behavior. If the purpose did not fit either purpose, we coded “Neither.”
Assessment versus treatment	We identified the primary purpose of the experiment and noted each as either assessment or treatment. The purpose of the experiment was assessment if the experiment implemented an evaluation that may contribute to a subsequent intervention within the same article or for future studies. The purpose would not be to change behavior but compare behavior or responding under some parameter or variable. The purpose of the experiment was treatment if the independent variable was purposefully manipulated and a dependent variable was measured to determine whether behavior changes in the desired direction. There may be one intervention or multiple interventions that lead to behavior change.
Intervention	If the purpose of an experiment was treatment, the intervention being implemented was noted.
Overall outcome	We identified the overall outcome of the experiment and noted each as either efficacious or effective. An outcome was efficacious if it demonstrated a phenomenon or answered a research question under tightly controlled conditions, using arbitrary or nonapplied stimuli or preparations. The main goal would be to answer a research question or demonstrate its occurrence but not to lead to meaningful behavior change. An outcome was effective if it demonstrated a behavior change that was meaningful to the participant, caregivers, family, or other stakeholders. The experiment could be conducted in tightly controlled conditions, but the purpose and goal of the study is to lead to behavior change that is relevant to the individual's daily life.

*Note*: FR = fixed ratio; VR = variable ratio; PR = progressive ratio.

### Comparison with Gilroy, Kaplan, and Leader (2018)

The present review was designed to extend rather than directly replicate Gilroy, Kaplan, and Leader (2018). Several core methodological features were intentionally retained to support comparison across reviews. Specifically, we maintained the same conceptual focus on operant‐demand‐related applications by extracting variables such as the presence of single‐ or concurrent‐operant arrangements, demand functions, work output functions, closed or open economies, and unit pricing. We also retained PRISMA‐guided review procedures (Page et al., 2021), multidatabase searching, inclusion of both single‐case and group‐design studies, and participant eligibility criteria requiring a diagnosis of autism or a related developmental disability.

At the same time, several methodological updates were introduced to improve rigor, transparency, and scope. First, although the original keyword structure was preserved, the present review systematically searched each operant demand term independently (i.e., “progressive ratio,” “demand curve,” “open economy,” “closed economy,” and “unit price”) across databases rather than relying primarily on PR indexing. This modification was intended to improve sensitivity for studies that incorporated operant demand concepts without explicitly labeling procedures as PR assessments. Second, the present review expanded coding variables beyond those reported by Gilroy, Kaplan, and Leader (2018). In addition to primary operant demand variables, age, and diagnosis, we coded participant race, dependent variables, assessment versus treatment purpose, intervention context, and study outcomes. These additions were intended to better characterize the applied contexts in which operant demand analyses are currently being used. An additional component coded was the specific procedural method, defined as the way researchers manipulated price to vary reinforcement schedules. This variable was included to allow for comparisons across methodological approaches and to better evaluate the clinical applicability and construct validity of different operant demand metrics. Third, study management and screening procedures were updated through the use of the Rayyan systematic review platform (Ouzzani et al., 2016; https://www.rayyan.ai/), which permitted structured duplicate removal, blinded screening, and documentation of reviewer disagreements. Finally, aligning with the PRISMA guidelines, interrater agreement procedures were expanded across search, title and abstract screening, full‐text review, and article coding phases, with explicit reporting of agreement percentages.

### Interrater agreement

Interrater agreement (IRA) was calculated for all phases of the search and screening process across the search period of 2018–2025 and the hand search. For database searching, the first author and an independent scorer (i.e., the second author and a trained graduate student) each randomly selected one database that the other searched, and each independently searched it using the same parameters described above. For the hand search, the first author randomly selected three journals that a trained graduate student searched and independently searched them using the same procedure (i.e., identifying all articles published in the respective volumes and issues of each journal). IRA for the initial database search was completed for 40% of databases at 99.80% (range: 99.20%–100%), and the initial hand search was completed for 33.33% of journals at 100%. The following phases report the combined IRA across the database and hand searches. During the title and abstract search, the first and second authors independently reviewed each study for 61.29% of articles reviewed, with 54 disagreements, yielding 97.13% IRA. The authors then discussed each disagreement and reached a consensus on whether to include the article. In total, 93 articles were identified for the full‐text review. During the full‐text review, IRA was collected for 42.55% of articles, with eight disagreements, resulting in 80% IRA. One additional article was also marked as “maybe” by one of the reviewers. The authors then discussed the nine flagged articles (i.e., the disagreements and maybe) and reached consensus on whether to include them.

Before collecting IRA for data coding, the second author randomly selected an article using an online random‐number generator (random.org) and coded that article independently. Practice coding articles continued until all primary variables had 100% agreement across three different articles. The practice articles’ scores were not included in the overall IRA score. IRA data collection was completed using the same data collection form as the first author. Then, IRA was collected for 30% of randomly selected articles using exact IRA for each variable at the participant level. If the random number generated an article that had already been coded, a new number was selected until a noncoded article was identified. An agreement was scored if both raters scored the same answer for that specific variable. A disagreement was scored if one rater scored one answer and the other rater scored a different answer. Total IRA was calculated as the total number of agreements divided by the total number of variables coded, multiplied by 100, yielding an average agreement of 95.58% (range: 90%–100%). Disagreements between the first and second authors were discussed until they both agreed on an answer.

## RESULTS

The search and screening procedures yielded 22 articles published between March 2017 and October 2023 and eight articles between November 2023 and December 2025 that incorporated at least one operant demand concept in assessment and treatment research involving individuals with autism and those with related developmental disabilities. Figure 2 depicts the search process and results. The initial systematic search yielded a total of 5,168 records. We then removed duplicates, leaving 3,069 articles. After reviewing each article's title and abstract and resolving disagreements, we moved 93 articles to full‐text review. From the full‐text review, we identified 28 articles to include in the review. We then searched each of the included articles’ reference lists and included two additional articles in the final review. Thus, the current review included 30 articles comprising 31 experiments and 307 participants. Two of these participants were included in two experiments from the same article and were therefore counted twice.

Across the time span from Gilroy, Kaplan, and Leader (2018) to the current review, the number of publications increased steadily, from 22 articles through February 2017 to 52 articles through December 2025 (Figure 3). Numerous articles were published in the recent 8.5‐year period, suggesting promising growth in applied research targeting challenges among individuals with autism and those with related developmental disabilities using operant demand concepts. Most experiments were primarily assessment‐focused, although several also embedded operant demand analyses within treatment evaluations. Twenty‐three of 31 experiments (74.19%) were classified as primarily evaluative (e.g., assessing reinforcer value under varying economic conditions), seven (22.58%) primarily targeted decreasing challenging behavior, and one (3.23%) primarily targeted increasing functional skills. The sections below summarize the findings for each research question. To preserve alignment with the study's research questions while clarifying comparability with Gilroy, Kaplan, and Leader, findings within each research question are organized into (a) direct extensions of Gilroy, Kaplan, and Leader and (b) novel findings from the current review.

**FIGURE 3:**
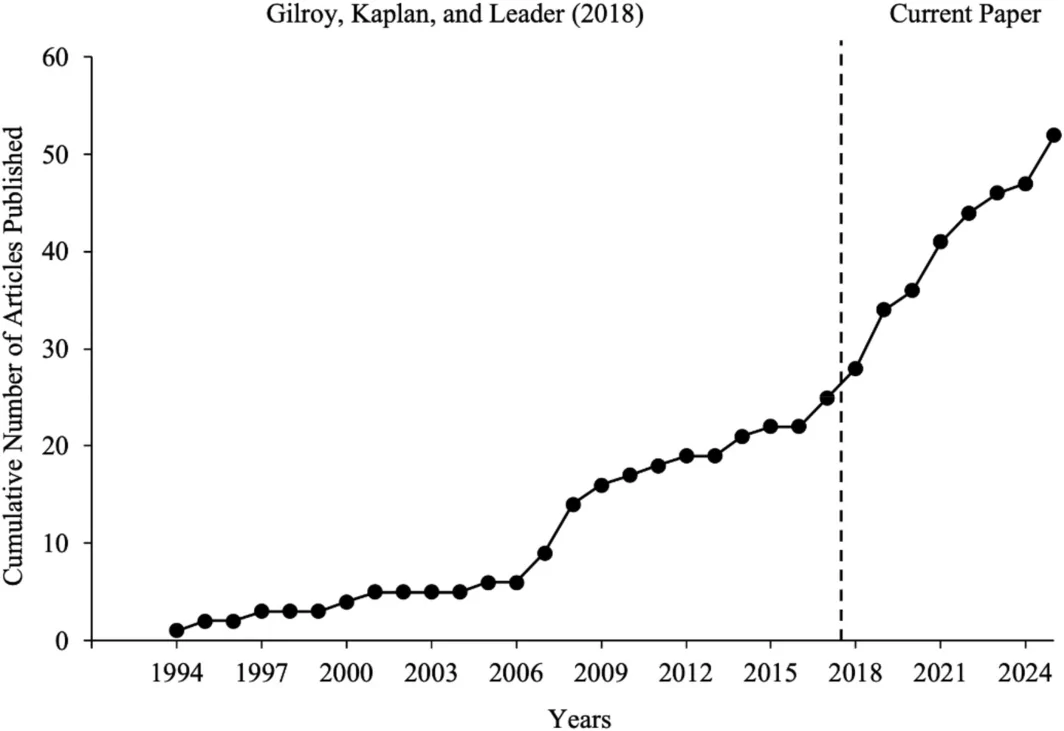
Cumulative number of articles published and included in Gilroy, Kaplan, and Leader (2018) and the current paper. The dashed line indicates the end of the search period of Gilroy, Kaplan, and Leader (2018) and beginning of the search period for the current review. Gilroy, Kaplan, and Leader (2018) ends in February 2017, and the current search is through December 2025.

### Research Question 1: Which operant demand concepts and procedures have been evaluated in the assessment and treatment literature involving individuals with autism and those with developmental disabilities?

#### Direct extensions of Gilroy, Kaplan, and Leader (2018)

Table 3 summarizes the operant demand concepts and procedures represented across each experiment included in the current review. Figure 4 provides a visual comparison of operant demand concepts between both reviews. Compared with Gilroy, Kaplan, and Leader (2018), there was an increase in the use of demand and work output functions in assessment and treatment research of individuals with autism and those with developmental disabilities. Specifically, 10 out of 31 experiments (32.26%) incorporated demand functions to model consumption as a function of price (i.e., response requirement or schedule density; e.g., Frank‐Crawford et al., 2018; Goldberg et al., 2017) and 14 experiments (45.16%) reported work output functions (e.g., Argueta et al., 2019; Kastner et al., 2023; Morris et al., 2025), quantifying the number of responses emitted at each price point. Five out of 31 experiments (16.13%) reported both (e.g., Gilroy et al., 2021; Lloveras et al., 2020). In most cases, demand and work output functions were derived from systematic manipulations of work requirements (e.g., PR schedule), with demand typically plotted as total reinforcer deliveries across prices and work output plotted as total responses per price. This represents a noticeable increase relative to the original review by Gilroy, Kaplan, and Leader, in which only seven of 22 studies (31.82%) reported demand functions and six of 22 studies (27.3%) reported work output functions. In the current review, demand analyses were used not only to evaluate reinforcer value but also to derive quantitative metrics of demand (e.g., demand intensity, elasticity, or breakpoints) that could inform clinical decisions about schedule thinning and treatment durability.

**TABLE 3:** Systematic review of experiments applying operant demand in research involving individuals with autism and those with developmental disabilities.

Study	Single‐ or concurrent‐operant [Number of participants]	Demand function	Work output function	Closed and open economy	Unit pricing	Procedural method		
						Price manipulation method	Within versus across sessions	Progression type [Step size]
Argueta et al. (2019)	1/0 [1]	–	Y	–	–	Increasing exchange‐production schedule	Across sessions	Arbitrary
Bonfonte et al. (2020)	3/0 [2]	Y	Y	–	–	Increasing FR requirement	Within session	Geometric [2]
Bonner et al. (2022)	3/0 [2]; 1/0 [1]	–	Y	–	–	Increasing exchange‐production schedule	Across sessions	Arithmetic [2]
Briggs et al. (2018)	0/1 [1]	–	–	–	–	Increasing FR requirement	Across sessions	Arbitrary
Castillo et al. (2022)	4/0 [4]	–	–	–	–	Increasing FR requirement	Within session	Arithmetic [2, 5]
Davis et al. (2017)	3/0 [2]	–	–	–	–	Increasing FR requirement	Within session	Arithmetic [1]
De Meyer et al. (2019)	55/0 [55]	–	–	–	–	Increasing FR and VR requirement	Within session	Arithmetic [1]
Falligant and Kornman (2019)	0/3 [1]	–	–	–	Y	Increasing token‐production and token‐exchange schedule	Across sessions	Arbitrary
Falligant et al. (2020), Exp 2	0/2 [3]	–	–	–	Y	Increasing token‐production schedule	Across sessions	Arbitrary
Frank‐Crawford et al. (2021), Exp 2	6/0 [1]; 4/0 [4]	–	Y	–	–	Increasing FR requirement	Across sessions	Arbitrary
Frank‐Crawford et al. (2018)	0/4 [9]	Y	–	–	–	Increasing FR requirement	Within session	Arbitrary
Gilroy et al. (2019)	2/1 [1]	–	Y	–	Y	Increasing FR requirement	Within session	Arbitrary
Gilroy et al. (2021)	2/0 [3]	Y	Y	–	Y	Increasing FR requirement	Within session	Arbitrary
Goldberg et al. (2017)	252/0 [21]	Y	–	–	–	Increasing token‐production schedule	Within session	Arithmetic [10]
Goldman and DeLeon (2022), Exp 1	0/6 [2]; 0/5 [2]	Y	–	–	–	Increasing FR requirement	Within session	Arbitrary
Goldman and DeLeon (2022), Exp 2	0/3 [3]	Y	–	–	–	Increasing FR requirement and token‐production schedule	Within session	Arbitrary
Hangen et al. (2023)	3/0 [2]	–	–	–	–	Increasing FR requirement	Within session	Arbitrary
Hoffmann et al. (2017)	2/0 [4]	Y	Y	–	Y	Increasing FR requirement	Within session	Geometric [2]
Kastner et al. (2025)	1/0 [1]	–	Y	–	Y	Increasing FR requirement	Across sessions	Arbitrary
Kastner et al. (2023)	2/0 [3]	–	Y	–	–	Increasing FR requirement	Across sessions	Arithmetic [1]
Kronfli et al. (2020)	2/0 [6]	Y	Y	–	–	Increasing FR requirement	Within session	Arithmetic [1]
Lambert et al. (2024)	552/0 [69]	–	–	–	Y	Increasing FR requirement	Within session	Arithmetic [3]
Leon et al. (2021)	2/0 [5]	Y	–	–	Y	Increasing FR requirement	Within and across sessions	Arithmetic [1]
Levins and Gilroy (2025)	1/0 [3]	–	Y	–	Y	Increasing token‐production schedule	Across sessions	Arbitrary
Lloveras et al. (2020)	2/0 [7]	Y	Y	–	–	Increasing FR requirement	Within session	Geometric [2]
Lloyd et al. (2021)	23/0 [23]	–	–	–	–	Increasing FR requirement	Within session	Arbitrary
Morris et al. (2025)	98/0 [49]	–	Y	–	–	Increasing FR requirement	Within session	Geometric [2]
Romani (2025)	2/0 [3]	–	–	–	–	Increasing FR requirement	Within session	Arbitrary
Rooker et al. (2022)	1/0 [9]	–	–	–	–	Increasing FR requirement	Within session	Geometric [2]
Russell et al. (2018)	3/0 [3]; 2/0 [2*]	–	–	Y	–	Increasing FR requirement and token‐production schedule	Within session	Arithmetic [1]
Taylor et al. (2025)	4/0 [2]	–	Y	–	–	Increasing FR requirement	Within session	Arithmetic [1]
Total	1,036/25 [309]	10	14	1	9			
Difference from Gilroy et al. (2018) review	+935 / +8	+3	+8	‐1	+6			

*Note*: The asterisk (*) denotes participants who were also included in the first single‐ or concurrent‐operant value. That is, these two participants were evaluated twice in this study. Exp = Experiment; Y = Yes, the component was present; – = No, the component was not present; FR = fixed ratio; VR = variable ratio.

**FIGURE 4:**
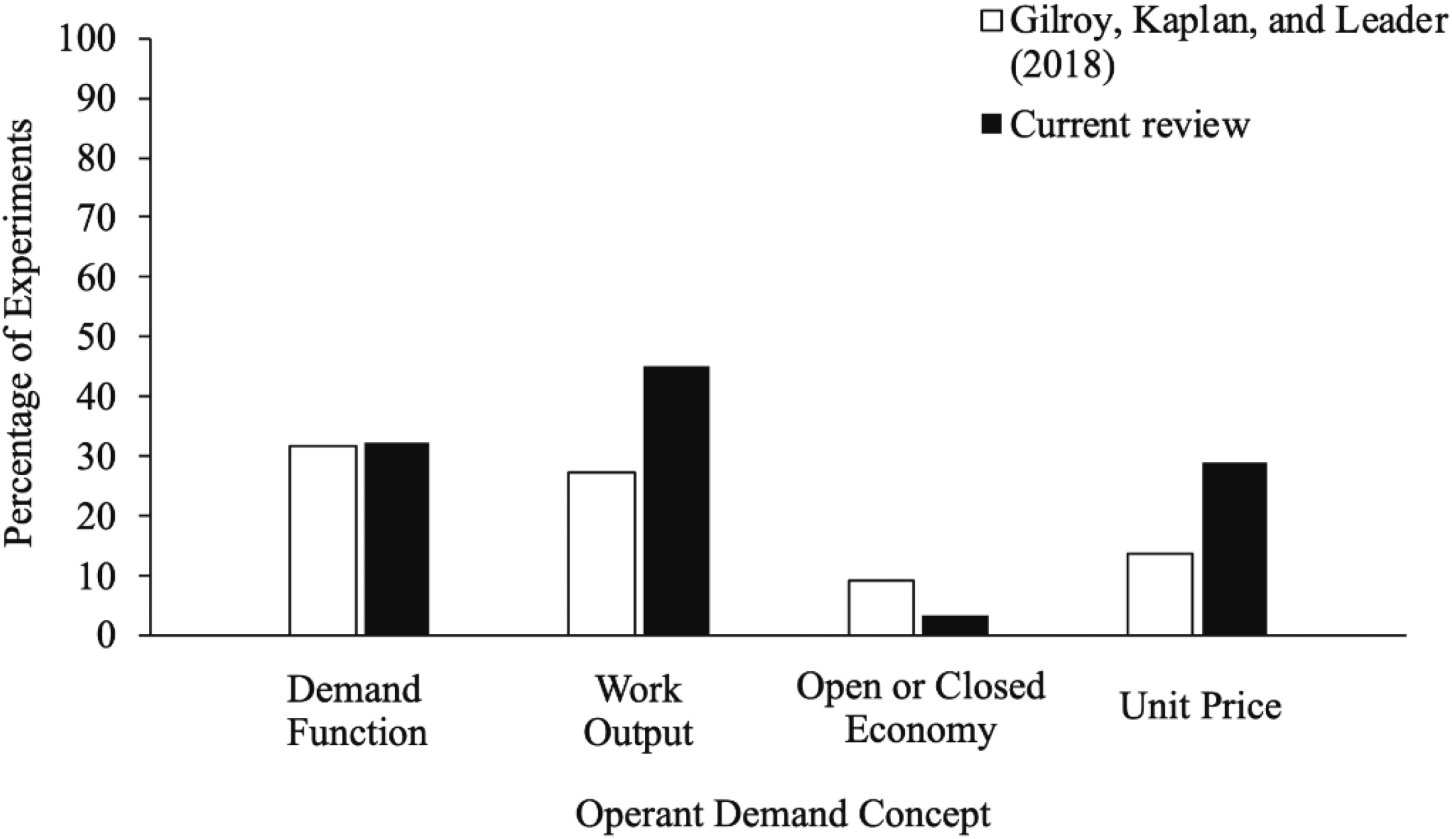
Comparison of operant demand concepts between both systematic reviews.

Despite the conceptual emphasis on open versus closed economies in behavioral economics, very few applied studies have evaluated this concept. Only one of the 31 experiments (3.23%; Russell et al., 2018) manipulated presession access to backup reinforcers within a token economy, enabling comparison of conditions in which reinforcement was effectively open (e.g., free access to edibles before sessions) versus closed (e.g., edibles restricted to within sessions). Under these conditions, responding maintained by edible reinforcement was disrupted by presession access, whereas responding maintained by tokens remained relatively stable. The current review identified only this one study compared with the two open and closed economy studies identified in Gilroy, Kaplan, and Leader (2018), suggesting that this concept has not expanded in the applied literature despite its translational promise.

Conversely, the use of unit price has increased substantially in recent years. In Gilroy, Kaplan, and Leader (2018), unit pricing appeared in only three out of 22 studies (13.64%). In the current review, nine of 31 experiments (29.03%) explicitly used unit‐price formulations, typically by expressing unit price as responses per unit of reinforcement (e.g., responses per token, responses per minute of access). These studies (e.g., Falligant et al., 2020; Gilroy et al., 2019, 2022; Leon et al., 2021) used unit price to compare reinforcers while both response requirement and reinforcer magnitude varied, equated conditions that differed across multiple dimensions (e.g., brief access at low cost vs. longer access at higher cost), and designed token economies that maintained approximately stable unit prices across phases while increasing absolute response requirements.

In the current review, across all experiments, researchers implemented a total of 1,036 single‐operant and 25 concurrent‐operant reinforcer assessments. Single‐operant arrangements remained the predominant format, replicating the pattern reported by Gilroy, Kaplan, and Leader (2018; e.g., Bonfonte et al., 2020; Kastner et al., 2023). Concurrent‐operant arrangements were less common but appeared in several studies examining choice or substitutability (e.g., Briggs et al., 2018; Frank‐Crawford et al., 2018).

#### Novel findings from the updated review

The expanded coding procedures also enabled a more detailed analysis of the specific procedures used to manipulate price and generate operant demand metrics such as breakpoint, P_*MAX*_, and O_*MAX*_. Across the 31 experiments, the most common price‐manipulation method was systematic increases in response requirements, typically implemented with PR or increasing fixed‐ratio (FR) schedules. For example, 22 of 31 experiments (70.97%) used increasing FR response requirements to manipulate price. These arrangements were typically used to evaluate reinforcer persistence under increasing work requirements and to identify transition points at which responding shifted from relatively inelastic to elastic patterns (e.g., Gilroy et al., 2021; Lambert et al., 2024). Other studies manipulated price through token‐economy‐based arrangements, including token‐production, exchange‐production, or token‐exchange schedules (see Hackenberg, 2018, for a review). Six experiments (19.35%) used these procedures (e.g., Argueta et al., 2019; Falligant & Kornman, 2019; Falligant et al., 2020), extending traditional response‐effort manipulations into generalized conditioned‐reinforcement and token‐economy contexts commonly used in applied settings (Kim et al., 2022). One experiment (3.23%) manipulated both FR and variable‐ratio (VR) response requirements (De Meyer et al., 2019), whereas two experiments (6.45%) combined increasing FR response requirements with token‐production schedules (Goldman & DeLeon, 2022, Experiment 2; Russell et al., 2018), suggesting limited but emerging procedural diversity in how price has been manipulated in applied operant demand research.

Most studies manipulated price within sessions rather than across sessions. Specifically, 21 out of 31 experiments (67.74%) arranged price increases within sessions, nine experiments (29.03%) manipulated price across sessions, and one experiment (3.23%) included both to directly compare the two approaches (e.g., Leon et al., 2021). Substantial variability was also observed in the progression type used to increase price. Arbitrary progressions were the most common progression type, occurring in 15 out of 31 experiments (48.39%), indicating that many studies selected individualized or clinically convenient response increments rather than adhering to standardized arithmetic or geometric scaling procedures. Arithmetic progressions were observed in 11 experiments (35.48%), whereas geometric progressions appeared in five experiments (16.13%). All studies involving geometric progression used a step size of two (e.g., 1, 2, 4, 8, 16 …), whereas the most common arithmetic progression increased by one response per step (e.g., 1, 2, 3, 4 …). Overall, these findings highlight substantial variability in the procedural methods used to manipulate price across applied operant demand studies.

### Research Question 2: In which assessment and treatment areas and contexts have these concepts been applied?

#### Direct extensions of Gilroy, Kaplan, and Leader (2018)

Table 4 summarizes the treatment areas and contexts represented across experiments included in the current review. The majority of the experiments (23 out of 31 experiments; 74.19%) were primarily assessment focused, with operant demand concepts used to refine measurement of reinforcer efficacy rather than to directly evaluate treatment plans. In many of these studies, operant demand analyses extended traditional preference assessments by showing that highly preferred items under low‐cost conditions did not always maintain responding under higher costs, thereby providing a more treatment‐relevant picture of reinforcer performance (e.g., Frank‐Crawford et al., 2018; Kronfli et al., 2020).

**TABLE 4:** Systematic review of assessment and treatment areas and contexts.

Study	Dependent variable	Intervention	Increase or decrease in behavior	Assessment versus treatment	Efficacy versus effective
Argueta et al. (2019)	Simple‐operant task responses	None	Neither	Assessment	Efficacy
Bonfonte et al. (2020)	Simple‐operant task responses	None	Neither	Assessment	Efficacy
Bonner et al. (2022)	Challenging behavior	Positive reinforcement of alternative behavior	Decrease	Treatment; assessment	Effective; efficacy
Briggs et al. (2018)	Challenging behavior; preference selection	FCT and schedule thinning	Decrease	Treatment	Effective
Castillo et al. (2022)	Academic responses	None	Neither	Assessment	Efficacy
Davis et al. (2017)	Simple‐operant task responses	None	Neither	Assessment	Efficacy
De Meyer et al. (2019)	Simple‐operant task responses	Continuous reinforcement, partial reinforcement, and stretching the ratios	Neither	Assessment	Efficacy
Falligant and Kornman (2019)	Preference allocation (not selection)	None	Neither	Assessment	Efficacy
Falligant et al. (2020), Exp 2	Preference allocation (not selection)	None	Neither	Assessment	Efficacy
Frank‐Crawford et al. (2021), Exp 2	Challenging behavior	Accumulated reinforcement	Decrease	Treatment; assessment	Effective; efficacy
Frank‐Crawford et al. (2018)	Challenging behavior; Preference selection	None	Neither	Assessment	Efficacy
Gilroy et al. (2019)	Challenging behavior	FCT and EXT	Decrease	Treatment; assessment	Effective; efficacy
Gilroy et al. (2021)	Academic responses; preference selection	None	Neither	Assessment	Efficacy
Goldberg et al. (2017)	Preference selection; simple‐operant task responses	None	Neither	Assessment	Efficacy
Goldman & DeLeon (2022), Exp 1	Preference selection	None	Neither	Assessment	Efficacy
Goldman & DeLeon (2022), Exp 2	Physical activity	Token economy	Increase	Treatment	Effective
Hangen et al. (2023)	Simple‐operant task responses	None	Neither	Assessment	Efficacy
Hoffmann et al. (2017)	Preference selection; simple‐operant task responses	None	Neither	Assessment	Efficacy
Kastner et al. (2025)	Challenging behavior	FCT and delay‐and‐denial tolerance training	Decrease	Treatment	Effective
Kastner et al. (2023)	Challenging behavior	DRA with fixed and incrementing reinforcing durations during schedule thinning	Decrease	Treatment	Effective
Kronfli et al. (2020)	Preference; simple‐operant task responses	None	Neither	Assessment	Efficacy
Lambert et al. (2024)	Simple‐operant task responses	None	Neither	Assessment	Efficacy
Leon et al. (2021)	Simple‐operant task responses	None	Neither	Assessment	Efficacy
Levins and Gilroy (2025)	Academic responses; Preference selection	None	Neither	Assessment	Efficacy
Lloveras et al. (2020)	Academic responses; preference selection	None	Neither	Assessment	Efficacy
Lloyd et al. (2021)	Simple‐operant task responses	None	Neither	Assessment	Efficacy
Morris et al. (2025)	Simple‐operant task responses	None	Neither	Assessment	Efficacy
Romani (2025)	Simple‐operant task responses	None	Neither	Assessment	Efficacy
Rooker et al. (2022)	Challenging behavior; simple‐operant task responses	FR 1, PR, and EXT	Decrease	Treatment; assessment	Effective; efficacy
Russell et al. (2018)	Academic responses	None	Neither	Assessment	Efficacy
Taylor et al. (2025)	Simple‐operant task responses	None	Neither	Assessment	Efficacy

*Note*: FCT = functional communication training; EXT = extinction; DRA = differential reinforcement of alternative behavior; FR = fixed ratio; PR = progressive ratio.

#### Novel findings from the updated review

A few experiments applied operant demand concepts to challenging behavior and treatment durability. Seven out of 31 experiments (22.58%) primarily focused on decreasing challenging behavior, often in the context of function‐based interventions (e.g., functional communication training [FCT] with schedule thinning or delay‐and‐denial tolerance training) for escape‐ or attention‐maintained behavior. Specifically, operant demand analyses were used to examine how treatment durability varied as reinforcement schedules were thinned (e.g., Bonner et al., 2022; Briggs et al., 2018; Frank‐Crawford et al., 2021; Gilroy et al., 2019; Kastner et al., 2023, 2025) and how arranging high‐rate operant tasks with potent alternative reinforcers can disrupt even treatment‐resistant automatically maintained self‐injury through response and reinforcer competition (Rooker et al., 2022). In contrast to Gilroy, Kaplan, and Leader (2018), in which most operant demand applications were confined to an assessment context, the present review includes several experiments in which operant demand analyses directly informed treatment decisions.

Across the 31 experiments, several other recurring application contexts emerged. These included token economies and generalized conditioned reinforcers in which demand and unit price were used to evaluate token efficacy, compare tokens with primary reinforcers, or design token‐economy schedules that maintained engagement under increasing cost (e.g., Bonner et al., 2022; Falligant & Kornman, 2019; Falligant et al., 2020; Goldman & DeLeon, 2022; Levins & Gilroy, 2025). Demand analyses were also used to evaluate reinforcer efficacy across a range of stimulus types and conditions including tangibles or edibles (Castillo et al., 2022), different types of social interaction (Davis et al., 2017), and high‐ versus low‐tech stimuli across varying durations of access (Hoffmann et al., 2017). Recently, these applications have also expanded to the study of social reinforcement and social motivation, including analyses of the reinforcing efficacy of social interaction and sensitivity to social rewards in children who are neurotypical and children with autism (e.g., Morris et al., 2025; Taylor et al., 2025).

### Research Question 3: Which populations are most commonly included in applied operant demand research?

#### Direct extensions of Gilroy, Kaplan, and Leader (2018)

Table 5 summarizes the participants’ demographics across the experiments included in the current review. Across the 307 participants in the 31 experiments, many of the participants had multiple diagnoses (*n* = 130; 42.35%), with autism as the most frequent single diagnosis (*n* = 66; 21.50%). At the experiment level, 28 of 31 experiments (90.32%) included participants with a diagnosis of autism, with many of these also reporting co‐occurring intellectual developmental disability, attention‐deficit/hyperactivity disorder, or specific genetic syndromes (e.g., Down syndrome). Age data indicated that participants were predominantly children and adolescents under the age of 18 (*n* = 225; 73.29%). Ages spanned from early preschool years to late adolescence (e.g., Briggs et al., 2018; Morris et al., 2025), with some including adults or mixed‐age samples (e.g., Hoffmann et al., 2017; Rooker et al., 2022). Several group designs (e.g., De Meyer et al., 2019; Goldberg et al., 2017; Lloyd et al., 2021) included broader age ranges and larger samples. Collectively, these findings suggest that although operant demand concepts have expanded across diagnostic profiles and study designs, the literature remains concentrated in populations of children with autism.

**TABLE 5:** Individual participant demographics in systematic review (*N* = 307).

Diagnosis	Counts (*n*)	Total percentage (%)
Autism	66	21.50
Intellectual disability	16	5.21
Down syndrome	2	0.65
Smith‐Magenis syndrome	2	0.65
Attention‐deficit/hyperactivity disorder	1	0.33
Asperger's syndrome	1	0.33
Pervasive developmental disorder not otherwise specified	1	0.33
Multiple diagnoses	130	42.35
Unknown	88	28.66
Age Ranges		
Total children (under 18)	225	73.29
Total adults	6	1.95
Unknown	76	24.76
Race		
White	92	29.97
Black	34	11.07
South Asian	2	0.65
American Indian or Alaska Native	1	0.33
Multiracial	3	0.98
Unknown	28	9.12
Missing information	147	47.88

*Note*: “Unknown” refers to a reported value that does not allow the researchers to categorize a variable into a specific class, and “Missing Information” refers to no information available.

#### Novel findings from the updated review

The expanded coding procedures in the present review permitted examination of participant demographic representation and reporting practices. Many experiments included in the current review did not report participants’ race (*n* = 147; 47.88%). These findings suggest that despite recent growth in applied operant demand research, demographic reporting remains inconsistent across experiments, which is consistent with the broader behavior‐analytic literature (e.g., Bruzek & Witts, 2025; Jones et al., 2020).

## DISCUSSION

Several broad themes emerged in the current review. First, as mentioned, operant demand concepts are increasingly informing treatment decisions rather than serving exclusively as assessment tools, as reported in Gilroy, Kaplan, and Leader (2018). Second, operant demand concepts could clarify why treatments succeed or fail under increasing constraint (e.g., response requirements). Despite their common occurrence in applied and clinical settings (Samaha et al., 2016), preference assessments alone did not consistently predict treatment‐relevant reinforcer performance, particularly under conditions of increasing effort or constraint for edibles (Frank‐Crawford et al., 2018; Kronfli et al., 2020), but sufficient for social interactions (Davis et al., 2017). Moreover, although children with autism remain the predominant population studied, the expanding application of operant demand concepts to experiments with a wide range of developmental and diagnostic profiles suggests broader clinical relevance, with some including adults or mixed‐age samples (e.g., De Meyer et al., 2019; Hoffmann et al., 2017; Lambert et al., 2024; Lloyd et al., 2021). Last, there was substantial variability in procedural methods used to manipulate price, highlighting the need for more standardized approaches to operant demand assessment. In the sections that follow, we focus on the first two themes, discuss their implications for research and clinical practice, and highlight their clinical utility and adoption of operant demand.

### Operant demand concepts are increasingly informing treatment, not just assessment

One of the most notable developments since Gilroy, Kaplan, and Leader (2018) is the growing use of operant demand concepts not only to measure reinforcer value but also to directly inform treatment selection and refinement. Several studies examined how reinforcer efficacy changed as response requirements increased, yielding clinically meaningful predictions about when and why preferred stimuli would fail to maintain behavior (Frank‐Crawford et al., 2018; Gilroy et al., 2021). Others applied demand analyses to identify reinforcers that would support durable performance during function‐based interventions, such as FCT with schedule thinning (Briggs et al., 2018; Gilroy et al., 2019; Kastner et al., 2025). One study (Rooker et al., 2022) demonstrated that arranging high‐rate alternative responding with highly preferred edible reinforcers could suppress treatment‐resistant, automatically maintained self‐injury, a subtype historically characterized by insensitivity to reinforcement‐based interventions (Hagopian et al., 2015, 2017). These findings suggest that operant demand analyses may reveal treatment effects that might otherwise be obscured in traditional functional analyses or noncontingent reinforcement schedules. Collectively, this literature suggests operant demand analyses can not only function as a method for quantifying reinforcer value but also have the potential to provide a decision‐making framework capable of guiding intervention design and anticipating treatment challenges before they emerge. For example, demand analyses may reveal that a reinforcer identified as highly preferred during a preference assessment shows steep elasticity as response requirements increase (Bonner et al., 2022; Frank‐Crawford et al., 2018; Kastner et al., 2023). This means that the reinforcer may be unlikely to support durable performance during schedule‐thinning in interventions such as FCT. In such cases, clinicians could proactively modify treatment by selecting reinforcers with more inelastic demand, adjusting unit price, or introducing alternative reinforcers to promote persistence.

### Operant demand concepts may clarify why treatments succeed or fail under increasing constraint

A second major theme involves the role of operant demand concepts in clarifying treatment durability, defined as the degree to which intervention effects maintain across changes in time, stimulus conditions, reinforcement conditions, or other relevant environmental variables (i.e., schedule thinning; Mitteer, 2025). Across multiple experiments, treatment outcomes were shown to depend not only on the presence of reinforcement but also on the elasticity of demand for the reinforcer used during the intervention (Bonner et al., 2022; Gilroy et al., 2019; Kastner et al., 2023; Lambert et al., 2024). Reinforcers characterized by more inelastic demand were more likely to support durable treatment effects during schedule thinning or extinction. In contrast, reinforcers with more elastic demand frequently failed to sustain responding as response requirements increased.

Kastner et al. (2023), for example, demonstrated that systematically increasing reinforcement magnitude in conjunction with higher response requirements reduced challenging behavior and supported more efficient progression toward terminal thinning goals. Similarly, demand analyses in Bonner et al. (2022) and Gilroy et al. (2019) identified predictable points at which responding would degrade under lean schedules. This type of information can be used to optimize thinning schedules and avoid treatment disruptions (e.g., relapse). Even in studies focused on assessment, patterns of elasticity and work output revealed why some reinforcers maintain responding in structured settings but fail in more naturalistic environments where competing alternatives or requirements are unavoidable (e.g., Russell et al., 2018).

More recent studies have also extended operant demand analyses to the study of reinforcer persistence and extinction. For example, Lambert et al. (2024) conducted a randomized trial and demonstrated that operant demand metrics such as demand intensity and elasticity influenced response persistence and extinction bursts under extinction conditions. That is, the more reinforcer individuals “consumed” before extinction, the less persistent and disruptive their responding became during extinction. Similarly, Romani (2025) used a PR reinforcement schedule to identify higher and lower quality reinforcers and showed that reinforcers maintaining responding under greater response requirements (i.e., higher quality) were associated with greater resurgence during extinction procedures. This finding implies that high‐quality reinforcers may initially strengthen treatment effects but practitioners should carefully plan reinforcement thinning and maintenance because these reinforcers may also increase the likelihood of resurgence when reinforcement stops.

Clinically, these findings suggest that operant demand analyses may be particularly useful for anticipating and mitigating treatment relapse, a persistent threat to the long‐term maintenance of behavior‐analytic interventions (Kimball et al., 2023). Treatment relapse, including resurgence during reinforcement thinning of an alternative behavior (Epstein, 1985; Lattal et al., 2017), renewal across contexts (Saini & Mitteer, 2020), or reinstatement following lapses in treatment integrity (Franks & Lattal, 1976; Podlesnik & Shahan, 2010), often occurs under conditions characterized by increasing effort, lean reinforcement, or competing alternatives, which are the conditions modeled by operant demand arrangements. Measures such as demand elasticity and work output may therefore function as quantitative indicators of relapse vulnerability, identifying reinforcers unlikely to sustain behavior as treatment demands increase. To better inform treatment durability, future research should examine dynamic changes in demand over time, as demand is likely to shift with learning, repeated exposure, satiation, and accumulated treatment history. Future research should examine how open and closed economies and reinforcer substitutability interact with demand, as these economic conditions may alter the value of reinforcers and the elasticity of demand (Hursh & Silberberg, 2008).

### Implications for future practice and research

The findings of the present review suggest several implications for future practice and research. First, across both clinical and research contexts, a central implication is that operant demand analyses can extend reinforcer evaluation beyond static preference rankings by quantifying how reinforcer value changes under increasing response requirements or competing alternatives (Hursh, 1980; Hursh & Silberberg, 2008; Reed et al., 2013). In practice, incorporating demand and work output functions into reinforcer assessments can help clinicians select reinforcers that are more likely to maintain behavior during common treatment challenges (e.g., schedule thinning, increased task demands, reduced reinforcement density), anticipate when responding is likely to degrade, and adjust reinforcement parameters (e.g., response requirement, magnitude, or unit price; Bonner et al., 2022; Gilroy et al., 2019; Kastner et al., 2023, 2025; Lambert et al., 2024; Romani, 2025).

At the same time, the field would benefit from more consistent and decision‐relevant reporting practices. Although the present review suggests a modest increase in studies reporting elasticity‐related metrics such as P_*MAX*_ and O_*MAX*_ relative to Gilroy, Kaplan, and Leader (2018), most studies continue to rely on visual inspection of demand curves alone. Reporting elasticity metrics such as P_*MAX*_ and O_*MAX*_ provides a theoretically grounded indicator of the response requirements under which a reinforcer is most likely to sustain behavior and can directly inform schedule‐thinning decisions and intervention planning (Gilroy, Kaplan, & Leader, 2018; Hursh & Silberberg, 2008).

In addition, greater attention to procedural variables may improve the interpretation of operant demand outcomes. The present review identified substantial variability in how price was manipulated across studies, suggesting that a more standardized framework for selecting and reporting price‐manipulation procedures may be needed. Consistent with this concern, Leon et al. (2021) reported poor correspondence between within‐session PR schedules and across‐session FR demand analyses, with responding persisting at substantially higher response requirements under within‐session arrangements. These findings suggest that procedural differences in how price is manipulated may produce meaningfully different estimates of reinforcer persistence and elasticity.

Finally, future research and practice would be strengthened by embedding operant demand analyses directly into treatment evaluation rather than limiting them to assessment contexts. Promising directions include using demand and elasticity analyses to select reinforcers for function‐based interventions (Bonner et al., 2022; Gilroy et al., 2019), manipulating unit price to maintain engagement while increasing response requirements (Falligant & Kornman 2019; Falligant et al., 2020; Kastner et al., 2025; Leon et al., 2021; Levins & Gilroy, 2025), and adopting longitudinal designs to examine how demands change over time with learning history, satiation, and treatment exposure (Lambert et al., 2024). Additionally, exploring other behavioral economic concepts (e.g., matching law, delay discounting) in applied research could also inform treatment development and maintenance of treatment effects. Expanding participant samples and settings, particularly to adolescents and adults and to vocational, group‐instruction, and community environments, would also improve external validity and clarify whether operant demand analyses and metrics predict durability and generalization across the service contexts.

### Clinical utility and adoption of operant demand analyses

A practical question raised by the present findings is whether the potential benefits of operant demand analyses justify the additional time, training, and analytic effort relative to preference assessments already common in applied practice. Operant demand analyses are unlikely to replace preference assessments but may provide additional clinical value when preference alone does not predict reinforcer efficacy under increasing response requirements, schedule thinning, or competing sources of reinforcement. In these contexts, demand intensity may characterize reinforcer efficacy under relatively low‐cost conditions, whereas elasticity, breakpoints, P_*MAX*_, and O_*MAX*_ may indicate how well and for how long reinforcers sustain responding as requirements increase (Bonner et al., 2022; Gilroy et al., 2019; Hursh & Silberberg, 2008; Lambert et al., 2024). Unit‐price analyses may further inform adjustments to reinforcer magnitude as response requirements increase, potentially supporting schedule thinning while reducing treatment disruption (Falligant et al., 2020; Gilroy et al., 2019; Kastner et al., 2025; Leon et al., 2021; Shawler et al., 2026). However, these metrics should be considered decision‐support tools rather than prescriptive indicators because demand may vary with contextual variables and brief assessments may not generalize to ongoing treatment (Lambert et al., 2024; Russell et al., 2018). Moreover, full demand‐curve analyses and quantitative modeling may be impractical in some service settings, suggesting that broader clinical adoption may depend on lower burden procedures and practitioner‐friendly tools that facilitate data collection, analysis, and interpretation (Gilroy, Kaplan, Reed, et al., 2018; Kaplan et al., 2019; Shawler et al., 2026).

### Limitations

Several limitations should be considered when interpreting the findings of this review. First, the search strategy was intentionally aligned with Gilroy, Kaplan, and Leader (2018) to support comparability, but the use of a finite set of keywords may have resulted in the omission of relevant studies. For example, some studies may have arranged conditions consistent with open or closed economies, substitutability, or unit‐price logic without explicitly labeling those procedures using operant demand terminology. As a result, these concepts may have been underrepresented in the current review.

Second, although the studies in this review were included based on predefined eligibility criteria and peer‐reviewed publication status, we did not apply a standardized methodological quality appraisal or risk‐of‐bias tool (e.g., assessment of experimental control, internal validity, or analytic rigor; see Gilroy et al., 2026, for discussion). As a result, variability across studies in design quality, reporting practices, and use of operant demand metrics (e.g., specification of price, elasticity, or P_*MAX*_) was not formally evaluated or weighed in the synthesis.

Third, we included only peer‐reviewed published studies, so our overall findings may be susceptible to publication bias given that we did not include any “gray” literature that could have included null findings (Tincani & Travers, 2019). Consequently, the current findings should be interpreted as descriptive of trends in the published literature rather than as evidence of differential effectiveness or methodological strength. Future systematic reviews would benefit from incorporating structured quality or risk‐of‐bias assessments and including unpublished research studies to better evaluate how study rigor influences reliability and overall findings.

Fourth, we used a relatively narrow set of exclusion criteria applied during article selection. Although these criteria were intended to maintain consistency with the applied focus and methodological standards of the prior review (Gilroy, Kaplan, & Leader, 2018), they may have limited representation of the broader range and variability of operant demand research conducted with individuals with autism and populations with related developmental disabilities, including translational or preliminary studies that may provide valuable information regarding emerging concepts and clinical applications.

## CONCLUSION

Overall, the reviewed studies demonstrate that operant demand concepts provide a powerful lens for understanding how reinforcer efficacy varies across contexts, tasks, and constraints within assessment and treatment research of individuals with autism and those with related developmental disabilities. As a field, applied behavior analysis has long relied on static measures of preference (e.g., DeLeon & Iwata, 1996); however, the present review suggests that dynamic, cost‐sensitive measures such as demand, elasticity, and unit price may complement existing practice and provide insight into the complexities of clinical practice. Additional research is needed to determine whether these measures can help identify durable reinforcers, anticipate schedule‐thinning challenges, and maintain treatment effects amid competing stimuli.

## AUTHOR CONTRIBUTIONS

The first author contributed to conceptualization, data curation, formal analysis, investigation, methodology, project administration, visualization, writing of the original manuscript draft, and review and editing of the manuscript. The second author contributed to conceptualization, formal analysis, investigation, methodology, visualization, writing of the original manuscript draft, and review and editing of the manuscript. The third author contributed to conceptualization, methodology, visualization, and review and editing of the manuscript.

## CONFLICT OF INTEREST STATEMENT

The author(s) declared no potential conflicts of interest.

## ETHICS APPROVAL

This research did not require IRB approval because it analyzed existing published data.

## Data Availability

All data generated or analyzed during this study are included in this manuscript.
